# Restoration of the Original Inhabitants: A Systematic Review on Fecal Microbiota Transplantation for Graft-Versus-Host Disease

**DOI:** 10.7759/cureus.23873

**Published:** 2022-04-06

**Authors:** Mohamad S. Alabdaljabar, Hafiz M Aslam, Sindhusha Veeraballi, Faizan A Faizee, Batool H Husain, Shumaila M Iqbal, Shahrukh K Hashmi

**Affiliations:** 1 College of Medicine, Alfaisal University, Riyadh, SAU; 2 Department of Hematology/Oncology, East Carolina University, Greenville, USA; 3 Internal Medicine, Saint Michael’s Medical Center, Newark, USA; 4 Internal Medicine, University of Pittsburgh Medical Center (UPMC), McKeesport, USA; 5 Department of Pathology, University of Illinois, Chicago, USA; 6 Department of Rheumatology, University of Cincinnati, Cincinnati, USA; 7 Blood and Marrow Transplant Division, Mayo Clinic, Rochester, USA

**Keywords:** hct, gvhd, microbiome, transplantation, microbiota, fecal

## Abstract

A compelling intervention to maintain healthy gut microbiota in graft-versus-host-disease (GVHD) is fecal microbial transplantation (FMT). To examine its role in GVHD, we conducted a systemic literature search using multiple electronic databases. Upon pooling of data, 79 patients from six studies and five case reports were included. Complete remission (CR) occurred in 55.9% of patients, and partial remission (PR) occurred in 26.5% of patients (82.4% overall response rate). A limited number of patients had treatment-related mortality (TRM), while few showed mild gastrointestinal (GI)-related and non-GI adverse effects. None of the studies directly examined the role of FMT in the prevention of GVHD. In conclusion, FMT seems to be a safe and effective strategy for the management of GVHD based on the current evidence. Due to the small number of patients evaluated and the absence of randomized data, one cannot portray FMT as a standard of care yet; however, the low toxicity along with the clinical improvement justifies this modality to be tested in a randomized fashion.

## Introduction and background

Microbial colonization nurtures a distinct group of commensal organisms that account for the endogenous flora of the intestine and constitutes the biological ecosystem, which is collectively known as microbiota. Microbiota and their genes are referred to as the microbiome [1,2]. This colonization starts during the intrauterine stage, and these microorganisms change with different factors (i.e., delivery method and breastfeeding status) and over time [3]. It also varies from one location to another within the intestine. A disequilibrium in microbiota homeostasis has been reported to have associations with a wide spectrum of diseases, including cancer, graft-versus-host disease (GVHD), diabetes mellitus, immune-mediated conditions such as inflammatory bowel disease, obesity, cardiovascular diseases, and psychiatric illnesses [4-9].

In recent years, there has been an expanding focus on the interplay between intestinal microbiota diversity and the outcome of graft-versus-host disease (GVHD) in hematopoietic cell transplantation (HCT) recipients. Back in the 20th century, it was discovered that mice with HCT, if kept in microbe-free conditions either by using antibiotics or isolation, developed acute GVHD, which was milder in severity [10,11]. Researchers also found that patients who acquire GVHD demonstrated the preponderance of Lactobacillales and Enterobacteriales and fewer Clostridia species. Knowing that Clostridia prevent inflammation in the intestine, it was observed that ingestion of 17 Clostridia isolates, which are extracted from human stool, improved the survival of mice with GVHD [12]. It was also inferred that in patients undergoing allogeneic HCT, the post-engraftment microbiota was remarkably different in dead patients when compared to patients who survived, who had an increased number of certain species such as γ-proteobacteria, including *Enterobacteriaceae* [13].

**Figure 1:**
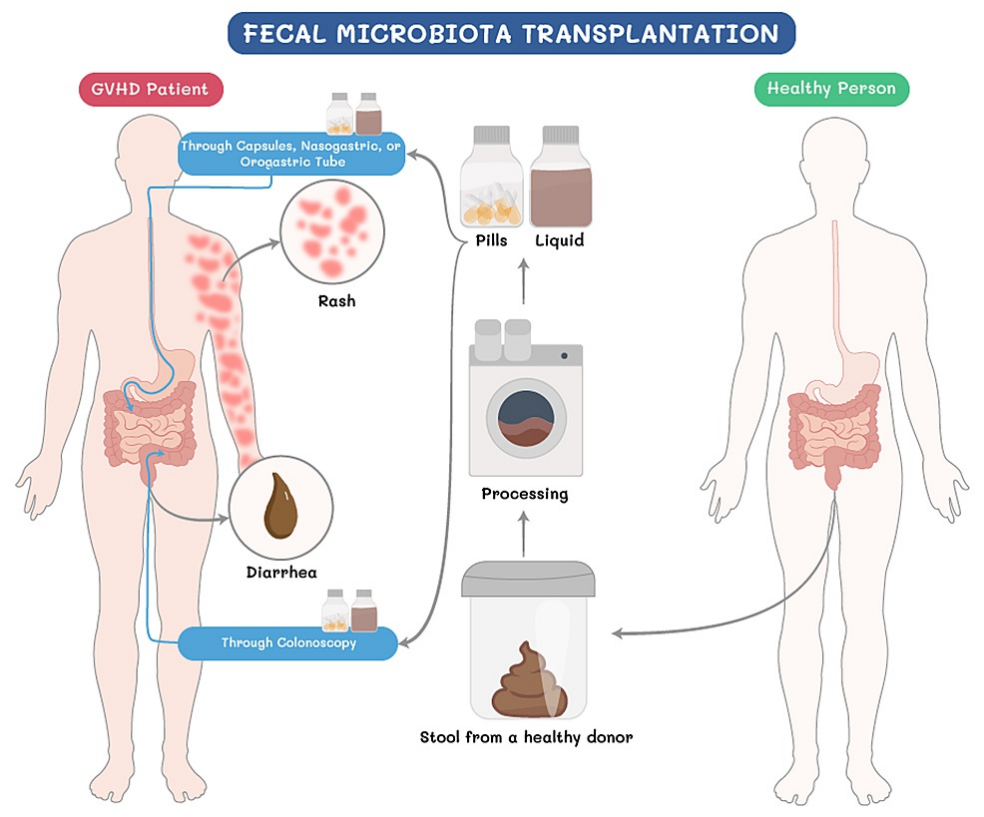
Fecal microbiota transplantation in graft-versus-host disease GVHD: graft-versus-host disease Image credits: Mohamad S. Alabdaljabar and Anas Idris

Exploiting the above concepts of microbiota and disease, fecal microbiota transplantation (FMT), which refers to the engraftment of a fecal suspension into the gastrointestinal (GI) tract to restore healthy microbiota [14], has been attempted in HCT for the treatment of GVHD (Figure 1). Besides the institutions that have innovative and pioneered approaches toward FMT, there is a general hesitancy among transplant professionals to recommend FMT as a therapeutic option for GVHD given the lack of approval by regulatory authorities and the lack of randomized clinical trials. Among HCT recipients, few studies focused on the role of FMT in *Clostridioides* *difficile* infections and on the treatment of GVHD. Herein, we aim to conduct a systematic review to study the role of FMT in GVHD treatment and prophylaxis.

## Review

Methodology

Search Strategy

The Preferred Reporting Items for Systematic Reviews and Meta-Analysis (PRISMA) guideline was used to conduct a systemic review of the literature. A literature search was conducted in PubMed/MEDLINE, Google Scholar, and Cochrane electronic databases for relevant articles using the following search string: “graft versus host disease,” “fecal microbiota transplantation,” and “hematopoietic stem cell transplantation.” The references of the retrieved articles were also manually checked for possible matching studies. The search was done between July 1 and 29, 2021.

Study Selection

We included articles that fulfilled the following criteria: (1) full, original studies focusing on FMT as a treatment/prophylaxis in GVHD patients, (2) studies in the English language, and (3) studies pertaining to GVHD in HCT only. As for the exclusion criteria, we excluded the following articles: (1) reviews, meta-analyses, book chapters, and animal studies, (2) studies on FMT used for non-GVHD conditions, or (3) articles from non-peer-reviewed journals.

Titles and abstracts were used to screen the articles to see whether they match our inclusion and exclusion criteria. All articles matching our search string were retrieved and carefully examined. Studies that matched all the inclusion criteria were analyzed in this systematic review.

Data Extraction

The included articles were reviewed by at least two authors independently. Data were extracted according to a predefined table looking for certain parameters, including publication year, study type, sample size, indication for bone marrow transplantation (diagnosis), GVHD type, route and donor of FMT, FMT outcome, and adverse effects. Any missing parameter in any of the included articles was replaced with “N/A.” Throughout this process and whenever an issue arises, a consensus was reached with the help of the corresponding author.

Results

**Figure 2:**
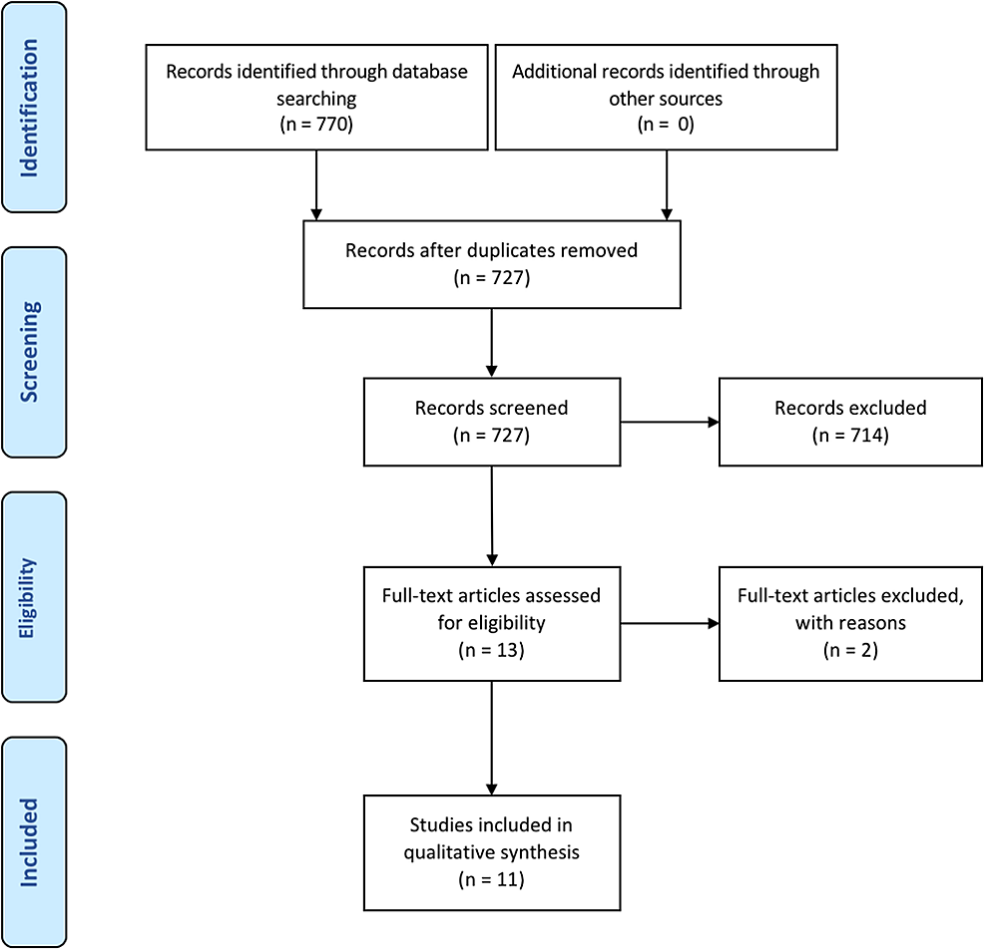
PRISMA flow diagram

Using our search string, 770 records were identified, of which 43 records were excluded due to duplication. A total of 727 studies were screened based on title and abstract. After screening, 714 studies were filtered out because they did not match our selection criteria. Thirteen studies were assessed, of which only 11 were found to meet our selection criteria. Upon pooling of data, 87 (79 underwent FMT) patients from six prospective/retrospective studies and five case reports/series were included in the final analysis (Figure 2). Our results are summarized in Table 1 in chronological order. Complete remission (CR) occurred in 55.9% of patients, and partial remission (PR) occurred in 26.5% of patients, which is equivalent to an 82.4% overall response rate in treating GVHD. CR and PR were not clearly defined in most of the included studies, so we used the authors’ descriptions in each study to categorize patients into these two groups. The last study by Goeser et al. [34] was not included in the calculation of CR and PR since the individual response was not delineated in the published manuscript.

Most of the articles were descriptive studies. Case reports/series and prospective studies come on top of the list with 5/11 studies in each category. The overall sample size was small for all the studies, which ranged between 4 and 27. All of the included patients underwent allogeneic HCT due to a wide variety of reasons, most commonly due to leukemia. Although our search string was liberal, using the aforementioned keywords and without any restrictions, we could not find any study that assessed the use of FMT prophylactically against GVHD; however, some studies [10] did mention it as a potential prophylactic tool, which will be discussed in the next section. All the included studies (11/11) focused on acute GVHD (aGVHD), and only two have also included some patients with chronic GVHD (overlap syndrome). In addition to gut GVHD, some patients had involvement of other organs; however, most of these studies focused on the improvement of GI GVHD as an outcome. The most common route for FMT appears to be using upper GI routes (e.g., upper GI endoscopy), but other routes including lower GI routes and oral capsules were also used to perform FMT. As for the number of FMT cycles, in most of the studies, patients have had 1-2 cycles up to seven cycles, as reported by Kaito et al. [15]. Moreover, studies have used related and unrelated donors with almost a 50% overall chance. Of note, since FMT is not yet approved for the treatment of GVHD, most of these studies used it after failing other lines of treatment, obtaining approval from their institution’s ethical committee, and/or obtaining informed consent from the patient/s.

When it comes to the safety of FMT and by looking at our data pool, FMT does not seem to be directly related to serious adverse effects. The adverse effects were mainly GI-related (abdominal pain/distention, nausea, regurgitation), but one of the studies [14] reported on non-GI adverse reactions, including infections, anemia, thrombocytopenia, and paroxysmal atrial fibrillation. Noteworthy, most of the studies have reported that FMT went uneventfully, and patients did not have major complications post-FMT.

Discussion

Multiple factors come into play in decreasing the variability of bacterial flora in an HCT patient, including administration of chemotherapy and/or radiotherapy, utilization of pre-HCT and peri-HCT antimicrobials, and nutritional status. Toll-like receptors (TLRs) are present on the mucosal surface of the intestines that modulate immune protection and also maintain the equilibrium of gut microbiota [16]. Murine models exhibited the presence of a symbiotic factor, polysaccharide A of *Bacteroides fragilis*, that regulates immune tolerance by associating with TLRs and FOXP3, which is present on regulatory T-cells [17]. A study on mice demonstrated that the colonization of the small intestine with one commensal microbe (segmented filamentous bacterium (SFB)) provokes the production of CD4 T helper cell 17 (Th17) [18]. This may suggest that intestinal microbes do play an important role in mucosal immunity. The interplay between all of these components makes us wonder about the potential application of FMT in patients who are prone to having their gut flora disturbed and a possible subsequent GI-related complication (i.e., GVHD patients).

On that basis, studies were done, and still going, to investigate and answer this question. In addition to what we presented, other preliminary data show promising results for the treatment of GVHD using FMT [19-21]. Another example of preliminary data was reported by Shouval et al. [22]. In their study, FMT capsules were given orally throughout the course in steroid-resistant/steroid-dependent acute GVHD patients. The administration of 15 courses of FMT capsules to seven patients with intestinal acute GVHD was done after extracting microbiota from a healthy unrelated donor. Few patients developed severe bacteremia, which was found, after metagenomic sequencing, to be unrelated to the FMT course. One of the patients had partial improvement, which was evident by a decrease in methylprednisolone dose by 0.8 mg/kg. FMT capsules were well tolerated by patients. Prior to the FMT course, four out of seven patients demonstrated *E. coli* dominance; however, bacterial diversity increased after the administration of FMT capsules. Three out of seven patients were alive; three died from the complications of acute GVHD, while one who was alive post-FMT died after invasive aspergillus infection. Overall, two patients achieved complete resolution following the FMT course, suggesting a crucial step in treating patients with GI GVHD. All of these data support the idea of treating GI GVHD with FMT.

When it comes to prevention by FMT, studies are still lacking. We could not identify studies focusing on that goal in specific. However, Jenq et al. [10] have shown promising results in using FMT prophylactically to improve GVHD outcomes. They identified the dynamics between microbial diversity and its effects on survival rates in patients with GVHD. They found that *Blautia* abundance was associated with an improvement in the overall survival rate (p < 0.001) in the entire cohort. After adjusting for graft source (umbilical cord grafts) and antibiotic exposure, it was found that the abundance of *Blautia* is associated with an almost 80% reduction in GVHD-related mortality (hazard ratio: 0.18; 95% CI: 0.05-0.63; p = 0.007) and 70% reduction for the need to use a systemic steroid to treat GVHD (hazard ratio: 0.3; 95% CI: 0.14-0.64; p = 0.002). The limitation of the study was that the sample was collected from one institution, and the causality was unknown. Taking the hypothesis of changes in microbial diversity and the prognosis of GVHD further, DeFilipp et al. [23] performed a pilot study on 13 subjects, before allogeneic HCT and 27 days post-HCT, who received FMT capsules from healthy donors. Patients tolerated FMT well with the exemption of one patient who experienced abdominal pain. One of the patients died of acute GVHD. The analysis of stool was performed, and urinary 3-indoxyl sulfate levels established a change in the diversity of the microbiome. The study revealed that the empiric FMT post-allogeneic HCT is a safe and feasible measure that can result in a significant change in the microbiota of the recipients.

There are several important points that are worth discussing in regard to FMT in GVHD and HCT recipients for that matter. First, although it seems to be effective, the safety is still questionable. Most of the studies have reported minimal to no serious adverse effects; however, many patients have eventually died in the long term. This is not to say that FMT is not safe; rather, it is to highlight the complexity of assessing safety in a population (i.e., HCT recipients) who are prone to develop serious complications regardless of FMT. Infections come on top of the list; the subsequent bacteremia and/or sepsis that happened in many patients is somewhat concerning. Since HCT recipients are already more prone to have such consequences, this concept is worth exploring in the future to better delineate whether FMT is related to short- and long-term adverse effects, including infection. The lack of standardized approaches to preparing and screening fecal samples is another issue that should be kept in mind as we move forward to advance FMT. Another point that would be important to investigate is the effects of FMT on other components of GVHD (i.e., skin) and not only the GI tract. Moreover, what we have achieved so far is promising, but certainly, we need studies that are better in both aspects of quality (i.e., randomized clinical trials) and quantity (i.e., more studies on a bigger scale). In addition to that, and although it was implied, efforts should also focus on conducting studies that can explore possible options to apply FMT as a prophylactic measure to prevent GVHD development. A recent single-arm, multicenter study on 25 patients with acute myelogenous leukemia (AML), post-intensive chemotherapy and antibiotic treatment, was done to study the effect of autologous FMT (AFMT) on microbiota restoration and dysbiosis resolution [24]. Chemotherapy resulted in microbiota dysbiosis and domination of pro-inflammatory species. In this study, AFMT was successfully used to restore intestinal microbiota diversity. Interestingly, these results showed a decrease in cumulative incidence of aGVHD. The reported cumulative incidence of aGVHD post-AFMT was 22% [24], which suggests an improvement from what has been previously reported in allogeneic HCT recipients with an aGVHD cumulative incidence of 34.6% [25]. These promising results may suggest a possible role for FMT as a preventive measure against GVHD post-HCT.

**Table 1:** The use of FMT for the treatment of GVHD GVHD: graft-versus-host disease; GI: gastrointestinal; FMT: fecal microbiota transplantation; CR: complete remission; PR: partial remission; AE: adverse effects; AML: acute myeloid leukemia; MDS: myelodysplastic syndrome; ALL: acute lymphoblastic leukemia; CML: chronic myeloid leukemia; HAL: hybrid acute leukemia; WAS: Wiskott-Aldrich syndrome; OMF: osteomyelofibrosis; NHL: non-Hodgkin lymphoma; HL: Hodgkin lymphoma; MM: multiple myeloma; ID: inherited diseases; MPD: myeloproliferative disorder; MF: myelofibrosis; T-PLL: T-cell prolymphocytic leukemia; Th: thalassemia; SEE: severe aplastic anemia; RD: related donor; URD: unrelated donor *This paper reports on two immunocompromised cases in which FMT was used to treat chronic diarrhea. One of the two patients was diagnosed with acute GI GVHD; thus, we used it as the only candidate on our table, since the other patient had diarrhea for another reason. ^The last study was not included in calculating the percentages for total CR and PR since individual responses were not reported.

Study	Year	Study type	Sample size	Diagnosis	GVHD type	Route (number of FMT cycles)	Donor	CR (% total)	PR (% total)	Possible related AE
Kakihana et al. [14]	2016	Pilot study, prospective	4	AML	GI, acute, steroid-resistant/steroid-dependent	Nasoduodenal tube (×2, except 1 patient ×1)	RD	3 (75%)	1 (25%)	GI symptoms, anemia, thrombocytopenia, hypoxia, paroxysmal atrial fibrillation, lower gastrointestinal bleeding, cholestatic liver damage, transplant-associated thrombotic microangiopathy
Spindelboeck et al. [26]	2017	Case series	3	AML, MDS	GI, acute, steroid-resistant, grade IV	Colonoscopy into the terminal ileum and cecum (×1, ×2, ×6)	RD, URD	2 (66.67%)	1 (33.33%)	Infections
Qi et al. [27]	2018	Pilot study, prospective	8	ALL, AML, CML, HAL, CML	GI, acute, steroid-resistant	Nasoduodenal (×2 or ×1)	URD	5 (62.5%)	3 (37.5%)	No severe AE
Kaito et al. [15]	2018	Case report	1	ALL	GI, acute	Oral capsule (×7)	RD	-	1 (100%)	N/A
Zhong et al. [28]	2019	Case report	1*	WAS	GI, acute	Nasojejunal (×2)	URD	1 (100%)	-	No AE
Biernat et al. [29]	2020	Case report	2	AML, OMF	GI, acute	Intranasal tube (×3, ×4)	URD	1 (50%)	1 (50%)	N/A
Goloshchapov et al. [30]	2020	Randomized clinical trial	FMT: 19 (CTL: 8, total of 27)	ALL, AML, MDS, NHL, HL, MM, CML, ID	FMT: GI: acute: 15, chronic (overlap syndrome): 4 CTL: GI, acute	Gastroduodenoscope 3, nasointestinal 7, ingested capsules 17	URD, RD	FMT: 9 (47%) CTL: 1 (13%)	FMT: 9 (47%) CTL: 4 (50%)	N/A
van Lier et al. [31]	2020	Prospective, single-arm	15	AML, MDS, HL, NHL, MPD/MF	GI, acute, steroid-resistant/steroid-dependent	Nasoduodenal (×1)	URD	10 (66.67%)	0	Infections, discomfort of the nasoduodenal tube, transient abdominal distention, cramps, nausea, regurgitation
Mao et al. [32]	2020	Case report	1	MDS	GI, acute	Oral capsule (×2)	URD	1 (100%)	-	None
Bilinski et al. [33]	2021	Prospective multicenter study	14	AML, CML, MDS, ALL, MM, HL, SEE	Acute/chronic	Nasoduodenal (×1, except in three patients ×2)	RD, URD	6 (42%)	2 (14.3%)	Septic shock, sepsis, norovirus-mediated gastrointestinal tract infection
Goeser et al. [34]	2021	Retrospective	11	AML, MDS, T-PLL, Th	GI, acute	Oral capsules or nasojejunal tube (×1 or ×2)	RD, URD	N/A	N/A	Abdominal pain, transformation of peristalsis, vomiting
Total	-	-	79	-	-	-	-	38 (55.9%)^	18 (26.5%)^	-

## Conclusions

The abovementioned studies vouch for the safety and effectiveness of FMT in the treatment of aGVHD and shed the light on a promising area that is yet to be thoroughly understood. The translation aspects of these studies indicate that the establishment of gut diversity is, probably, the key mechanism behind the success of FMT. With the currently available evidence, one cannot portray FMT as a standard of care yet; however, the low or potentially absent toxicity along with improvement in survival justifies this modality to be tested in a randomized fashion.

Future studies should focus on understanding what microbial species are associated with positive and negative outcomes post-FMT, especially with respect to antimicrobial exposures in GVHD patients. More psychosocial studies are also needed to understand how receptive HCT patients and clinicians are to the concepts, costs, and preparation of FMT and its availability in different HCT centers around the globe. Moreover, while there is a lot of emphasis on the evaluation of human bacteriome, there is a paucity of studies in mycobiome and virome. Future studies in HCT recipients should focus on virome, since the phage-bacteriome interaction in the human gut is a critical determinant of health. Lastly, it is important to evaluate the effects of FMT on GVHD as a whole and not only gut-related symptoms. This promising area needs more rigorous studies to establish causation and, if so, to incorporate FMT in the guidelines to improve the management of GVHD patients. Until randomized data is available, we strongly encourage the transplant community to enroll patients in innovative trials utilizing FMT as this may be a safe and effective strategy for both prevention and treatment of GVHD.
